# Factors associated with prenatal care and HIV and syphilis testing during pregnancy in primary health care

**DOI:** 10.11606/s1518-8787.2019053001205

**Published:** 2019-09-12

**Authors:** Cláudia Helena Soares de Morais Freitas, Franklin Delano Soares Forte, Angelo Giuseppe Roncalli, Maria Helena Rodrigues Galvão, Ardigleusa Alves Coelho, Sonia Maria Ferreira Dias

**Affiliations:** I Universidade Federal da Paraíba. Departamento de Clínica e Odontologia Social. João Pessoa, PB, Brasil.; II Universidade Federal do Rio Grande do Norte. Programa de Pós-Graduação em Saúde Coletiva. Natal, RN, Brasil; III Universidade Estadual da Paraíba. Departamento de Enfermagem. Campina Grande, PB, Brasil; IV Universidade Nova de Lisboa. Instituto de Higiene e Medicina Tropical. Lisboa, Portugal

**Keywords:** Prenatal Care, HIV Infections, diagnosis, AIDS Serodiagnosis, Syphilis, Congenital, diagnosis, Infectious Disease Transmission, Vertical, prevention & control, Health Care Quality, Access, and Evaluation

## Abstract

**OBJECTIVE:**

To evaluate the factors associated with HIV and syphilis testing during pregnancy in Brazil.

**METHODS:**

This was an ecological study covering all Brazilian municipalities evaluated by the second cycle of the National Program for Access and Quality Improvement in Primary Care, 2013-2014. The dependent variables were based on prenatal care access: prenatal care appointments, and HIV and syphilis tests during prenatal care. The independent variables were compared with demographic and social characteristics. Bivariate analysis was performed assessing the three outcomes with the independent variables. Variables with significant associations in this bivariate analysis were fit in a Poisson multiple regression analysis with robust variance to obtain adjusted estimates.

**RESULT:**

Poisson regression analysis showed a statistically significant association with the variables “less than eight years of study” [prevalence ratio (PR) = 1.31; 95%CI 1.19–1.45; p < 0.001] and “participants of the cash transfer program” (PR = 0.80; 95%CI 0.72–0.88; p < 0.001) for the outcome of “having less than six prenatal care appointments” and individual variables. A statistically significant association was found for “participants of the cash transfer program” (PR = 1.43; 95%CI 1.19–1.72; p < 0.001) regarding the outcome from the comparison between HIV testing absence during prenatal care and demographic and social characteristics. The absence of syphilis testing during prenatal care, and demographic and social characteristics presented a statistically significant association for the education level variable “less than eight years of study” (PR =1.75; 95%CI 1.56–1.96; p < 0.001) and “participants of the cash transfer program” (PR = 1.21, 95%CI 1.07–1.36; p < 0.001).

**CONCLUSIONS:**

The individual factors were associated with prenatal care appointments and HIV and syphilis tests in Brazilian pregnant women. They show missed opportunities for diagnosing HIV and syphilis infection during prenatal care and indicate weaknesses in the quality of maternal health care services to eliminate mother-to-child transmission.

## INTRODUCTION

Maternal syphilis and HIV infections are a global concern and important public health problems. The overall number of children living with HIV has decreased since antiretroviral medicines to HIV-positive pregnant women were provided (over 3.0 to 2.6 million)^1^. Mother-to-child transmission rate in Latin America and The Caribbean has decreased from 15% to 8%. The estimated coverage of antiretroviral therapy in HIV-positive pregnant women for mother-to-child transmission prevention between 2010 and 2015 increased by 52% to 88%^2^.

Nearly 1 million pregnant women are infected by syphilis worldwide^1^. A growing rate of 1.7 cases per 1,000 live births has been reported, and 22,000 cases of congenital syphilis were estimated in the Americas in 2015. In Brazil, the congenital syphilis rate per 1,000 live births was 6.49 in 2015, and HIV prevalence in females aged 15-49 was 0.4 (0.3–0.6)^2,3^.

Syphilis/HIV identification in pregnant women is essential for vertical transmission prevention and treatment. Prenatal care services are the entry point for the elimination of mother-to-child transmission. A combined approach to these diseases is recommended by the WHO (World Health Organization) for promoting better maternal and child health^4,5^.

Early detection and administration of suitable therapies are central interventions to prevent congenital syphilis^5^. Interventions to prevent mother-to-child HIV transmission include early access to quality prenatal care, HIV testing, counseling and antiretroviral therapy^6^ Despite the expansion of prenatal syphilis screening programs, many barriers limit maternal syphilis screening and treatment efforts around the world, and therefore their potential solutions^7,8^. Syphilis continues to be a major public health concern in developing countries.

In Brazil, the health policy for AIDS and congenital syphilis^9^ recommends specific actions and goals to improve dissemination control, including increasing HIV and syphilis test coverage during prenatal care. Several studies in hospitals and in Primary Health Care^10^ observed that HIV and syphilis test coverage has increased in all Brazilian regions. Despite the contributions, no nationwide studies in Primary Health Care have been found in Brazil.

Some studies have shown no difference in congenital syphilis and HIV incidence in areas with greater coverage of Family Health Strategy units^15^. In addition, they report failures in primary care^17^. These findings reinforce the importance of a national study assessing primary care quality^16,19,20^.

Although the Brazilian government has invested in the availability of prevention and treatment actions in this area, few national studies were conducted with pregnant women with access to prenatal care and adequately monitored for the number of appointments, HIV and syphilis testing, and counseling. This study aims to evaluate the factors associated with non-testing of HIV and syphilis during pregnancy in primary health care in Brazil.

## METHODS

### Study Area

This study used data from the 2^nd^ National Program for Access and Quality Improvement in Primary Care (2^nd^ PMAQ-AB cycle) in Brazil, an external evaluation cycle held between 2013 and 2014. This survey was conducted by the Brazilian Ministry of Health in 5,211 cities throughout the country, including the 27 state capitals, representing 93.5% of Brazilian cities. The 30,424 primary care teams that joined the program were evaluated, corresponding to almost 90% of all primary care teams in the country. Further details about the National Program for Access and Quality Improvement in Primary Care in Brazil have been previously reported^21,22^.

### Data Sources

Prenatal care, demographic and social characteristics data were obtained from the National Program for Access and Quality Improvement in Primary Care (PMAQ-AB) database, second external evaluation. More specifically, we analyzed the answers of users interviewed in Brazil in Module III – Interview with users in the health services, which investigates user’s perception and satisfaction regarding use and access.

### Study Population and Sample

The study population comprises the Brazilian public health system users assessed in PMAQ-AB. About 114,615 users were interviewed. This study population consisted of 13,020 female users who met the following criteria: not being assisted for the first time, not having spent more than 12 months without service, and with children up to 2 years old.

### Variables

The dependent variables were: performing prenatal care in the unit, primary health services, number of prenatal appointments, performing HIV and VDRL tests.

The criteria for assessing the prenatal care were based on the protocols of the Ministry of Health. These protocols recommend that prenatal care should begin by the 12^th^ gestational week^23^, with a minimum of six appointments during follow-up and performing HIV serological and Venereal Disease Research Laboratory (VDRL) tests.

The independent variables are related to demographic and social characteristics of women who had prenatal care in primary health services. These variables were obtained from a questionnaire, which included: age, race, formal education level, work and participation in the *Bolsa Fam*í*lia* conditional cash transfer program. These variables are representative of these women’s socioeconomic position and show how this position can affect the health service access distribution.

### Statistical Analysis

The three outcomes were initially analyzed according to the independent variables. The prevalence is shown in absolute and relative values of each category, and the effect was estimated through the prevalence ratios (PR) and their respective confidence intervals of 95% (95%CI). Variables with significant associations in this bivariate analysis were fit in a Poisson multiple regression analysis with robust variance to obtain adjusted estimates.

### Ethical Issues

This study was based on secondary data from publicly available datasets (http://dab.saude.gov.br/portaldab/ape_pmaq.php), and therefore did not require ethical approval. The individual data were obtained from the public database of the National Program for Access and Quality Improvement in Primary Care (PMAQ-AB), approved by the Ethics Committee of the Federal University of Rio Grande do Sul under the Protocol 21,904. Patient information was anonymous and de-identified prior to analysis.

## RESULTS

From the 13,020 women participating in this study, 76.4% (9,945) had prenatal care in the primary health services unit, while 23.6% (3,075) did not receive prenatal care in the service, and were therefore excluded from this study as they did not fit the analysis scope. The answers “I do not know” or “not answered” were also excluded.

Table 1 shows descriptive analysis results of the dependent variables compared with demographic and social characteristics. The women that had six or more prenatal appointments are mostly white, over 30 years old, with less than eight years of study and participant in the cash transfer program. Women who had up to five prenatal appointments were predominantly non-white, between 15 and 19 years old, with more than eight years of study and not participant in the cash transfer program. Significant differences regarding formal education and cash transfer program participants were observed.

**Table 1:** Bivariate associations between prenatal care, HIV and syphilis tests and maternal demographic and social characteristics. Brazil,2014.

Variable	Prenatal appointments (n = 8,238)		p	HIV test (n = 9,749)		p	VDRL test (n = 8,904)		p
		
	< 5 (%)	≥ 6 (%)		Yes (%)	No (%)		Yes (%)	No (%)	
Age (years)									
15–19	19.4	80.6	0.171	92.0	8.0	0.000	78.9	21.1	< 0.001
20–29	17.9	82.1		94.5	5.5		86.9	13.1	
≥ 30	16.8	83.2		96.5	3.5		91.8	8.2	
Race									
White	17.1	82.9	0.438	96.3	3.7	< 0.001	89.8	10.2	< 0.001
Non White	17.9	82.1		94.3	5.7		86.7	13.3	
Years of schooling									
≥ 8	20.5	79.5	< 0.001	92.0	8.0	< 0.001	38.2	52.3	< 0.001
< 8	16.7	83.3		96.0	4.0		61.8	47.7	
Work									
Yes	17,5	82.5	0.742	95.8	4.2	0.016	90.0	10.0	< 0.001
No	17,8	82.2		94.5	5.5		86.6	13.4	
Cash transfer programme									
Yes	16.2	83.8	< 0.001	93.7	6.3	< 0.001	86.0	14.0	< 0.001
No	19.2	80.8		95.9	4.1		88.7	11.3	

VDRL: Venereal Disease Research Laboratory

The coverage rate for the HIV testing was 94.8%. The percentual is higher in white women over 30 years old, with less than eight years of study and not a cash transfer program participant. Women that did not undergo the anti-HIV test were predominantly non-white, between 15 and 19 years old, with less than 8 years of study and a cash transfer program participant. Apart from work, all demographic and social characteristics resulted in significant associations. The syphilis testing coverage rate was 87.5%, and the bivariate analysis results were quite similar (Table 1) to the HIV testing. The outcome of performing tests for syphilis detection during prenatal care were significantly associated with all demographic and social characteristics.

Tables 2, 3 and 4 show Poisson regression results to the outcomes of prenatal care and the demographic and social characteristics. A statistically significant association with the variables “less than eight years of study” (PR = 1.31; 95%CI 1.19–1.45; p < 0.001) and “participants of the cash transfer program” (PR = 0.80; 95%CI 0.72–0.88; p < 0.001) was found in the outcome “having less than 6 prenatal care appointments” and individual variables.

**Table 2:** Results of Poisson multiple regression analysis for the outcome “having less than 6 prenatal care appointments” and individual covariates.

Covariates	Less than 6		Non-adjusted		Adjusted	
		
	n	%	PR (95%CI)	p	PR (95%CI)	p
Age (years)						
15–19	203	19.4	1			
20–29	839	17.9	0.93 (0.81–1.06)	0.143		
≥ 30	422	16.8	0.87 (0.75–1.00)	0.034		
Race/Color						
White	391	17.1	1			
Non-White	1,030	17.9	1.04 (0.94–1.16)	0,438		
Years of schooling						
≥ 8	971	16.7	1		1	
< 8	493	20.5	1.23 (1.12–1.36)	< 0.001	1.31 (1.19–1.45)	< 0.001
Work						
Yes	347	17.5	1			
No	1,117	17.8	1.02 (0.91–1.13)	0.371		
Cash transfer programme						
No	820	19.2	1		1	
Yes	642	16.2	0.84 (0.77–0.93)	< 0.001	0.80 (0.72–0.88)	< 0.001

**Table 3:** Results of Poisson multiple regression analysis for the outcome “do not perform HIV tests during prenatal care” and individual covariates.

Covariates	No HIV tests		Non-adjusted		Adjusted	
		
	n	%	PR (95%CI)	p	PR (95%CI)	p
Age (years old)						
15–19	97	8.0	1		1	
20–29	302	5.5	0.69 (0.55–0.86)	< 0.001	0.65 (0.52–0.82)	
≥ 30	106	3.5	0.43 (0.33–0.57)	< 0.001	0.37 (0.28–0.49)	
Race/Color						
White	104	3.7	1		1	
Non-White	386	5.7	1.53 (1.24–1.89)	< 0.001	1.36 (1.10–1.69)	
Years of schooling						
≥ 8	281	4.0	1		1	
< 8	223	8.0	1.97 (1.66–2.34)	< 0.001	1.91 (1.59–2.29)	
Work						
Yes	103	4.2	1			
No	402	5.5	1.30 (1.05–1.60)	0.016		
Cash transfer programme						
No	213	4.1	1		1	
Yes	290	6.3	1.53 (1.28–1.81)	< 0.001	1.43 (1.19–1.72)	< 0.001

**Table 4:** Results of Poisson multiple regression analysis for the outcome “do not perform tests for syphilis detection during prenatal care” and individual covariates.

Covariates	No syphilis test		Non-adjusted		Adjusted	
		
	n	%	PR (95%CI)	p	PR (95%CI)	p
Age (years old)						
15–19	231	21.1	1			
20–29	657	13.1	0.62 (0.54–0.71)	< 0.001	0.61 (0.53–0.70)	< 0.001
≥ 30	229	8.2	0.39 (0.33–0.46)	< 0.001	0.36 (0.30–0.43)	< 0.001
Race/Color						
White	260	10.2	1		1	
Non-White	824	13.3	1.30 (1.14–1.49)	< 0.001	1.19 (1.05–1.36)	0.008
Years of schooling						
≥ 8	669	10.4	1		1	
< 8	447	18.1	1.74 (1.56–1.95)	< 0.001	1.75 (1.56–1.96)	< 0.001
Work						
Yes	223	10.0	1			
No	894	13.4	1.34 (1.17–1.54)	< 0.001		
Cash transfer programme						
No	539	11.3	1		1	
Yes	576	14.0	1.24 (1.11–1.38)	< 0.001	1.21 (1.07–1.36)	0.001

The Poisson regression results only showed a statistically significant association of the variable “participants of the cash transfer program” (PR = 1.43; 95%CI 1.19–1.72; p < 0.001) regarding the outcome of HIV testing absence during prenatal care and demographic and social characteristics. Finally, Poisson regression results also showed a statistically significant association of the variables “less than eight years of study” (PR =1.75; 95%CI 1.56–1.96; p < 0.001) and “participants of the cash transfer program” (PR = 1.21; 95%CI 1.07–1.36; p < 0.001) when compared syphilis testing absence during prenatal care and demographic and social characteristics.

## DISCUSSION

This study assessed the individual determinants of HIV and syphilis testing access during prenatal care in Brazil. From the implementation of the *Rede Cegonha* (Stork Network) starting in 2011, the prenatal care provides rapid HIV and syphilis testing. Prenatal care is an important opportunity to diagnose these diseases, providing both prevention of mother-to-child transmission (PMTCT) and access of pregnant women and their partners to available care practices in order to reduce morbidity and mortality from these diseases in the adult population^24^.

The strategy for eliminating congenital syphilis was launched by the WHO in 2007^4^ and reinforced in 2012^6^, when the integration of the prevention of mother-to-child transmission of HIV and congenital syphilis program during prenatal care was recommended. The Pan American Health Organization’s goals^25^ are to reduce mother-to-child transmission of HIV (MTCT) to ≤ 2% and its incidence to ≤ 0.3 cases per 1,000 live births. In addition, a reduction in the congenital syphilis incidence to less than 0.5 cases per 1,000 live births. These targets have been adopted by the Brazilian Ministry of Health. Twenty-two (22) countries in the Americas reported a similar number in 2015 regarding achieving the goal of eliminating MTCT of HIV^2^.

In Brazil, the policy proposes an integration between maternal health care and childcare by the protocols of HIV and syphilis^9^ and the *Rede Cegonha*^26^ Program to prevent vertical transmission. The elimination of mother-to-child transmission of both HIV and syphilis requires early access to quality prenatal care for all pregnant women. Although most women in this study have had six or more prenatal care appointments, it did not ensure HIV and syphilis testing performance, which shows missed opportunity of prevention during prenatal care. The proportion found in this study may be underestimated by the difficulty in reporting this information, especially in women with lower education level. One study pointed out that some pregnant women or mothers may overestimate their performance of the appointments to demonstrate carefulness^27^. On the other hand, studies checking pregnant women’s health cards revealed problems in information recording, which may cause underreporting^27^.

A Brazilian cohort study using data from the pregnant woman’s health card, medical records and interviews showed that black or brown women with lower education level and those who attended public health services had less syphilis and HIV testing coverage, and these were the women with the highest prevalence of syphilis during pregnancy^30^. Another study revealed social inequalities in prenatal care. Non-white women with lower education level and family income start the prenatal care later and, when they have access, it is of poor quality^31^.

On the other hand, the number of prenatal appointments of six or more per pregnancy potentially reflects an increase in access to primary health care with the Family Health Program in Brazil^32^, and a more equitable health service use^33^ in areas covered by the program. Our findings showed that non-white younger women attend fewer prenatal appointments. An increase in the number of women with children under 20 years old that had syphilis during pregnancy was observed in the historical series of cases of congenital syphilis and syphilis in pregnant women from 2005 to 2017 in Brazil, suggesting a greater probability of having recent forms and higher transmission of syphilis in this age group^34^. This result is important for health management and action service implementation aimed at this vulnerable population.

Less-educated women and participants in the cash transfer program had the syphilis testing lowest coverage rate, while the HIV testing lowest coverage rate was among women participating in the cash transfer program. Some studies^35,36^ reported low education as a congenital syphilis and HIV perinatal infection predictor. This suggests missed opportunities for diagnosis and intervention among women with increased risk of vertical syphilis and HIV transmission.

We observed in this study that receiving the family financial assistance (*Bolsa Familia*) was insufficient to prevent inequalities in access to testing for these diseases. On the other hand, receiving family financial assistance was associated with a greater number of appointments, which suggests financial difficulties to access the testing within the primary care services. National studies indicate failures in prenatal care regarding syphilis and HIV control, with missed opportunities for diagnosis and treatment^13,26,32^.

Studies in other countries showed missed opportunities in testing of HIV and infection by syphilis^30^ during pregnancy^37^, and the importance of an integrated program to ensure that HIV and syphilis testing are offered and performed on all pregnant women^38,39^. In Brazil, structural problems in diagnostic support services and delays in result returning seem to be the main limitations for achieving 100% coverage of pregnant women for VDRL and HIV tests^13^.

Some weaknesses have been pointed out by the Ministry of Health, among which are: the increase in the discovery of cases due to the availability of rapid care tests; penicillin shortages; the reference to other levels attention by of the almost half of primary health care units, thus losing patient for not treating^3^.

The main challenge for congenital syphilis control is the timely diagnosis in pregnancy^40^. Congenital syphilis can be largely prevented with inexpensive pregnant mother penicillin treatment if/when correctly diagnosed^41^. Its vertical transmission cannot be prevented with measures adopted at birth, requiring the services to diagnose and treat infection during pregnancy. Regarding HIV, care measures can be adopted to avoid diagnosis only occurring late in pregnancy or upon admission for delivery^42^.

Some authors recommend the use of rapid tests carried out in health units for pregnant women with limited access to health services, which would allow a more timely intervention^8,43^. However, implementing rapid tests in primary care is still difficult ^19,20,21^.

This study has some strengths and limitations. A strength is the completeness of the survey with national coverage in primary health care from which the data were extracted. However, the findings must be interpreted with some limitation considerations.

This study presents a cross-sectional design analysis of data. However, it does not enable the determination of cause and effect. The data were collected from a non-probability convenience sample of patients. Moreover, a possible selection bias cannot be excluded regarding women included in this study, since only women who had prenatal care in the evaluated primary health care facilities were considered. Firstly, having a child up to two years old was used as a proxy for gestation, which excluded women who had fetal losses and miscarriages in this period. Secondly, a memory bias also due to children’s age limit. National studies evaluating the maternal reporting reliability and pregnant women’s cardiac records show that both methods have limitations^28^. Underreporting was observed in the use of health cards by the pregnant women such as incomplete cards and, in some cases, cards left at the services, which hinders interprofessional actions. Registry absence suggests non-performance of procedures.

Despite these study limitations, this large-scale study provides useful insights into prenatal care, and HIV and syphilis testing during pregnancy. Further studies are necessary to improve our understanding of the complex dynamics of mother-to-child transmission of both HIV and syphilis in this population.

Although there has been an increase in prenatal coverage, and that most pregnant women in this study had access to six or more prenatal care appointments, the findings show missed opportunities for diagnosing HIV and syphilis infection during prenatal care. This was associated with individual socioeconomic position, especially education level in Brazilian pregnant women and indicate weaknesses in the quality of maternal health care services to eliminate mother-to-child transmission.

Therefore, improving service organization is necessary to increase the syphilis and HIV control program effectiveness. Quality improvement in these programs could make a difference in reducing maternal and infant morbidity and mortality caused by both syphilis and HIV.
